# Extreme Temperatures, Hospital Utilization and Public Health Insurance Spending

**DOI:** 10.3389/ijph.2025.1607160

**Published:** 2025-02-12

**Authors:** Yusun Kim, Qing Miao, Ling Zhu

**Affiliations:** ^1^ Graduate School of Public Administration, Seoul National University, Seoul, Republic of Korea; ^2^ Department of Public Policy, Rochester Institute of Technology, Rochester, NY, United States; ^3^ Department of Political Science, University of Houston, Houston, TX, United States

**Keywords:** extreme temperature, public health insurance, public health spending, health expenditure, hospitalization, healthcare utilization, climate change, United States

## Abstract

**Objectives:**

This study examines the impact of extreme temperatures on hospital utilization and public health insurance program spending in a country with no universal health coverage.

**Methods:**

Using nationwide U.S. county-level panel data and a fixed effects model, we estimate the impact of annual variations in the number of hot and cold days on hospital utilization and medical reimbursements for low-income and elderly beneficiaries of public health insurance.

**Results:**

Our results show that extreme heat and mild cold increase medical reimbursements to low-income beneficiaries, while extreme cold increases benefit transfer to the elderly. We find that extreme temperatures have particularly stronger positive effect on hospital admission and inpatient care utilization among old and poor patients. The fiscal impact of extreme temperatures is greater in areas with more generous income eligibility criteria for public health insurance.

**Conclusion:**

The study advances our understanding of how extreme temperatures affect healthcare utilization of low-income and elderly populations and the roles public health insurance plays in supporting them from increasing weather risks. Our findings suggest that climate change can augment the financial burden on governments.

## Introduction

As climate change unfolds, extreme heat and cold events are increasing worldwide, posing growing risks to human health and causing substantial economic and welfare costs. The effects of extreme temperature on mortality [1–5] and morbidity [6–9] have been well documented by studies from both developed and developing countries. Earlier studies show that heat increases the use of hospital care and particularly emergency health services [6, 10–13], while cold may reduce hospital visits [14]. However, there remains limited understanding of how extreme weather conditions influence the use of different types of healthcare services, and the associated financial implications for both households and governments. Even less is known about how extreme temperatures affect government spending on public health insurance programs. In the U.S., Medicare and Medicaid enrollees are primarily low-income and elderly individuals, who are particularly vulnerable to extreme weather due to higher medical risks and limited capacity to manage environmental challenges [15] Therefore, it is crucial to understand whether these individuals access more medical services in response to extreme temperatures, and whether public health insurance program spending increases further due to extreme weather.

Governments in the U.S. play an important role in providing and financing health services. At the federal level, Medicaid and Medicare spending, when combined, accounts for the largest share of the federal outlay (24% and 1.18 trillion USD in 2019). State and local governments spend a considerable part (322 billion US dollars in 2020) of their total general expenditure on public health services and hospitals. Conceptually, there are multiple channels through which extreme temperatures can affect medical service utilization and public health spending. First, heat and cold may affect the demand for healthcare services by worsening certain health conditions, particularly among the vulnerable populations such as the elderly. Such adverse health impacts can raise utilization of healthcare services and related spending. Second, extreme heat and cold may decrease healthcare service utilization by disrupting infrastructures and transportation systems [16]. Extreme temperatures may also impact adequate service provision at hospitals if hospital infrastructures are damaged, such as through power outages. The situation can be more complicated when the healthcare system does not meet the increasing demand, causing delays and decreases in healthcare utilization [16]. In this paper, we explore several mechanisms, by estimating the net effects of heat and cold on hospital service utilization.

Our analysis is based on nationwide U.S. county level annual panel data from 2000 to 2019 and hospital level annual data from 2008 until 2019, leveraging the plausibly exogenous annual variations in the number of extreme hot and cold days within each county. First, we find that extreme heat increases overall hospital utilization (admissions, inpatient days, and emergency room visits), while cold reduces emergency room visits. Second, our study demonstrates that extreme heat and mild cold increase Medicaid benefit transfer payments, while extreme cold increases Medicare transfers. Third, we find that heat and cold induce a larger increase in Medicaid benefit transfers in areas with more generous public health insurance. We also find that the impact of extreme heat and mild cold on Medicaid transfers is more pronounced in counties with more elderly and low-income populations.

Our research contributes to different strands of literature on climate economics, public health policy, and public finance. First, this is one of the first studies to examine the fiscal implications of extreme temperatures for government spending on public health programs. No study has yet analyzed the impact of extreme weather conditions on public health insurance costs. Previous studies have documented how extreme weather shocks affect local economies, personal income, and other socioeconomic outcomes [17]. Meanwhile, other studies have investigated the effects of natural disasters such as extreme weather events on national and local governments finances including public spending and intergovernmental transfers [18–24], but only very few have examined the effects of disasters (hurricanes and flooding) on social safety net spending [23, 25]. This study fills the gap in the existing literature by focusing on the impact of extreme temperatures on public health insurance program spending through the mechanism of health service utilization, and also considering how these effects may vary depending on the generosity of the state-level Medicaid program.

A previous study reported larger increases in emergency hospital admissions associated with extreme cold than with extreme heat in England, showing a prominent effect among senior and socially deprived patients [8]. Nonetheless less is known about how extreme temperature may affect healthcare utilization among low-income and elderly populations covered by targeted public health insurance in a country without universal health coverage. Existing studies in the U.S. focus on the effect of extreme heat on hospital utilization in a specific city or state, which limits their external validity. For instance, several studies have shown the impact of extreme heat on hospital admissions in a single metropolitan city or a state [26–28]. Other studies focus on documenting the health impact of heat on specific population, such as the elderly [29–32] or pregnant females [33–35].

Therefore, the purpose of this paper is to present empirical evidence on the net effect of extreme temperatures on medical service utilization by patients covered by public health insurance and government expenditure on these programs in a developed country with no universal health coverage.

## Methods

### Data

First, to create the temperature variables, we obtain the station-level daily temperature data from the Global Historical Climatology Network of the National Climate Data Center (NCEI). Our sample time frame is from 2000 until 2019. We measure the daily temperature using a simple average between the maximum and minimum temperatures reported by the NCEI and map the weather stations to counties using the station’s latitude and longitude information. If a county contains multiple weather stations, we average their measures for each day following the previous literature [36]. This approach helps to minimize the impact of localized variations and outliers from individual stations, providing a consistent and reliable dataset for analysis. We merge our weather data with public medical transfer and hospital utilization data obtained from multiple sources. We aggregate the daily weather data to the county level for each year, measuring the number of days with temperatures falling in predefined bins and annual precipitation.

We use two separate samples depending on the outcome variable. In the hospital utilization sample, the unit of analysis is hospital-by-year, while in the medical transfer sample the unit is county-by-year. Our hospital utilization data is from the American Hospital Association Annual Survey, a yearly dataset that includes hospital level utilization records such as hospital admissions, inpatient day, emergency department and outpatient visits [37]. The final hospital-level sample is a strongly balanced annual panel of 5,558 hospitals in 2,457 counties from 2008 until 2019 (49,552 observations). We consider hospital utilization as a reasonable indicator of healthcare service utilization since hospital care represents the largest share of personal healthcare spending in the country.

**FIGURE 1:**
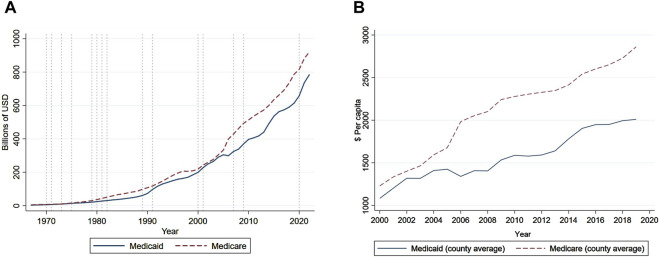
Historical trend of Medicaid and Medicare transfer (United States, 2021). Note: Panel **(A)** shows the trend in the national sum of annual Medicaid and Medicare transfer payments. Panel **(B)** plots the county-level annual average amount of transfer payments during the sample period from 2000 until 2019.

For the medical transfer analysis, we use a separate annual county-level sample (57,809 observations for 3,045 counties) from 2000 until 2019 on government payments through public medical programs from the Regional Economic Information System published by the Bureau of Economic Analysis. Medical transfer measures are government transfer payments made directly or through intermediaries to healthcare providers for services utilized by individuals covered by Medicare and Medicare. Public health insurance is an integral part of the U.S. healthcare system and finances a sizable share of medical service fees incurred by low-income or older Americans. Medicare benefit transfers are paid by the federal government, since Medicare is a federal program. These fees have been increasing rapidly as shown in Figure 1. Table 1 Panel A shows that benefit transfer (reimbursement of medical service fees from the government) to Medicaid beneficiaries accounts for 96% of total Medicaid expenditure in 2019. Medicare is primarily financed through the federal government’s general revenues and payroll taxes. State Medicaid programs are co-financed by the federal and state governments, while some states also require their local governments to share the non-federal portion of Medicaid expenditure.

**TABLE 1:** Medicaid enrollment and expenditures, selected years (United States. 2018).

Panel A. Medicaid enrollment, benefit and administrative expenditures							
Fiscal Year	Enrollment (Millions)	Benefit expenditures			Administrative expenditures		
		Federal (%)	State (%)	Total ($bn)	Federal (%)	State (%)	Total ($bn)
1970	14	53	47	4.9	50	50	0.2
1980	19.6	55	45	24	58	42	1.2
1990	22.9	56	44	68.7	57	43	3.5
2000	34.5	57	43	195.7	56	44	10.6
2010	54.5	68	32	383.6	55	45	17.9
2017	73.4	62	38	572.2	64	36	27.8
2019	75.1	62	38	611.7	62	38	27.7

Panel B. Medicaid benefit payments: share by beneficiary group and total amount						
Fiscal Year	Aged 65Y	Disabled *<*65Y	Children *<*20Y	Adults 20–64Y	Expansion Adults 20–64Y	Total ($ per enrollee)
2010	38.3	44.6	6.7	10.4	NA	40,705
2011	38.0	44.2	6.9	10.9	NA	41,434
2012	38.1	44.5	6.9	10.5	NA	40,013
2013	36.8	45.5	7.1	10.7	NA	41,084
2014	35.7	45.0	7.6	11.7	0.1	41,145
2015	34.2	45.7	8.0	12.2	0.2	42,001
2016	34.0	45.8	8.2	11.9	0.1	43,220
2017	33.6	45.4	8.5	12.5	0.1	44,870
2018	33.1	45.8	8.8	12.3	0.1	44,526
2019	32.7	45.9	8.9	12.5	0.1	45,337

Note: Enrollment count is in millions of persons, while benefit and administrative expenditures are in billions of US dollars ($bn). Figures are from “2018 Actuarial report on financial outlook for Medicaid” by the Office of the Actuary Centers for Medicare and Medicaid Services, United States Department of Health and Human Services.

Note: Payment shares by beneficiary group are indicated as percentages. 65Y indicates at or above 65 years of age; <65Y means less than age 65; <20Y stands for less than age 20; 20–64Y refers to between ages 20 and 64. All figures are from “2018 Actuarial report on financial outlook for Medicaid” by the Office of the Actuary Centers for Medicare and Medicaid Services, United States Department of Health and Human Services.

We also control for county-level socioeconomic and demographic characteristics that vary annually. These time-varying characteristics include the percentage share of Black, Hispanic or senior populations (from American Community Survey), unemployment rates (from the Bureau of Labor Statistics), and median personal income and weekly wage in the private sector (from the Bureau of Economic Analysis), all of which are aggregated to the county-annual-level. Table 2 shows the summary statistics of both hospital and transfer samples.

**TABLE 2:** Summary statistics of key variables (United States. 2019).

Variable	Obs	Mean	Std.Dev.	Min	Max
Panel A. County Transfer Sample					
Below 10°F	60,845	5.92	11.16	0	87.00
10–20°F	60,845	11	12	0	102
20–30°F	60,845	24	19	0	114
30–40°F	60,845	43.26	21.73	0	225.00
40–50°F	60,845	54.78	16.41	0	179.00
50–60°F	60,845	60.00	14.86	0	282.00
60–70°F	60,845	66.97	14.56	0	199.00
70–80°F	60,845	69.55	29.73	0	203.00
80–90°F	60,845	28.94	33.26	0	193.00
Above 90°F	60,845	0.66	4.27	0	120.00
Total transfer ($ per capita)	62,020	8,908	2,671	0	21,670
Medicare transfer ($ per capita)	60,998	2,107	750	127	6,777
Medicaid transfer ($ per capita)	60,998	1,571	823	0	9,011
Total transfer (log of $ per capita)	60,998	9.07	0.28	7.34	9.98
Medicare (log of $ per capita)	60,998	7.58	0.39	4.84	8.82
Medicaid (log of $ per capita)	60,988	7.23	0.55	1.74	9.11
Percent Aged	62,020	16.49	4.54	1.68	58.50
Percent Black	62,020	8.88	14.46	0	85.93
Percent Hispanic	62,020	8.04	13.11	0	97.54
Total population	62,020	97,747	315,215	55	10,100,000
Urban indicator	62,014	0.46	0.50	0	1.00
log of total employed	61,480	9.57	1.50	3.97	15.70
Average wage per capita, private sector	61,480	13.40	9.54	1.52	316.67

Panel B. Hospital Sample (logs)					
Hospital Admissions	49,552	7.75	1.54	0.69	11.92
Inpatient Days	49,552	9.69	1.47	0.68	13.54
Outpatient Visits	49,552	10.27	3.05	0	15.65
Emergency Room Visits	49,552	9.52	1.54	0	13.31
Medicaid Inpatient Days	49,552	7.62	2.10	0	13.22
Medicare Inpatient Days	49,552	8.78	1.50	0	12.76

Note: All fiscal variables are adjusted for inflation (constant 2010 U.S.dollars). Std.Dev. stands for standard deviation. “Min” stands for minimum and “Max” stands for maximum.

### Empirical Models

We examine the impact of extreme temperature, measured by the number of extreme hot and cold days, on a variety of health-related outcomes including hospital utilization and transfer payments through Medicare and Medicaid. We first model the effect of temperature on various hospital utilization outcomes using the following two-way fixed effect model as shown in Equation 1:

$$U_{i,c,t}={\Theta D}_{c,t,j}+{\alpha M}_{s,t}+{\Pi X}_{c,t-1}+\gamma i+\lambda t+\varepsilon_{i,c,t}$$ (1)

**FIGURE 2:**
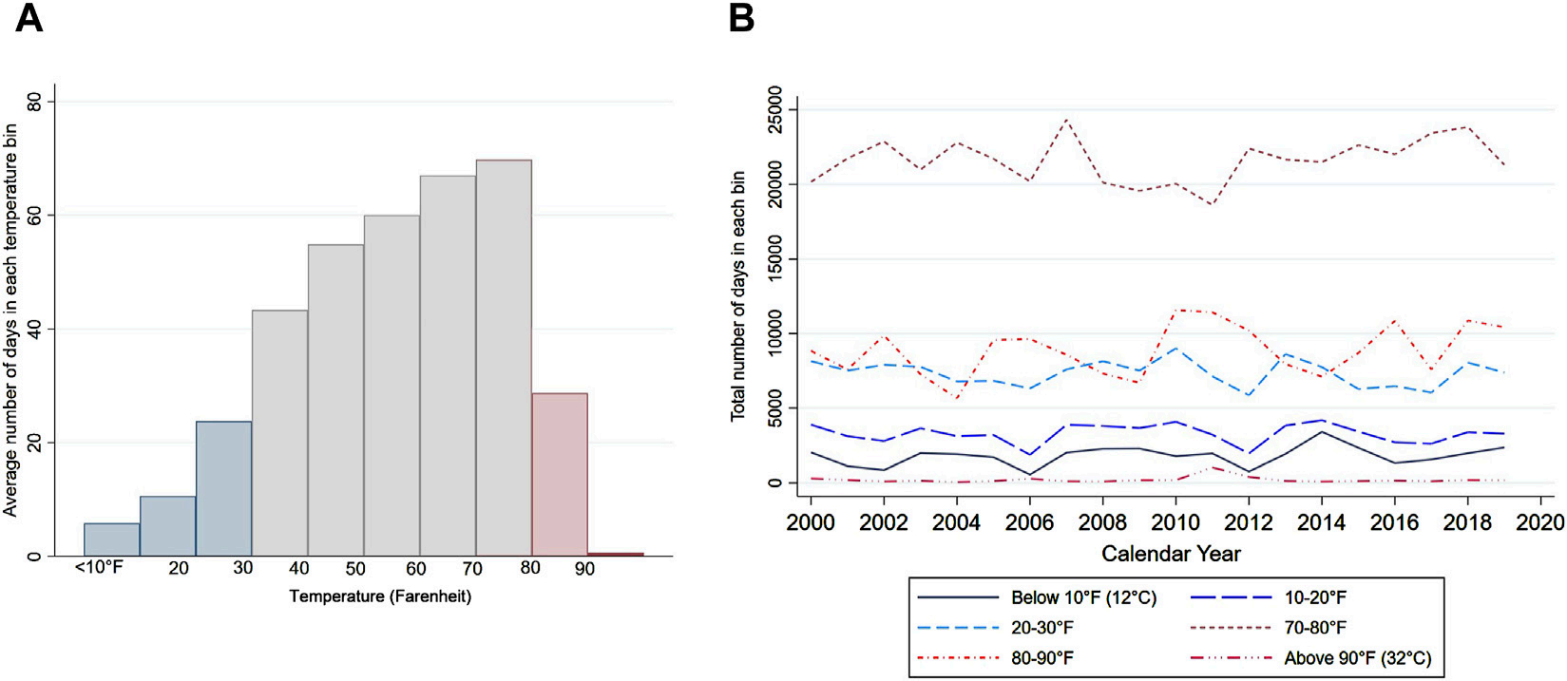
Distribution of daily mean temperatures (United States, 2020). Note: Panel **(A)** figure shows the distribution of daily mean temperatures across ten temperature bins. The horixontal axis denotes the temperature bin separated by 10°F (or 12.2°C). Panel **(B)** shows the national average number of days in each temperature bin over the sample period.

Where *U*_*i,c,t*_ is hospital utilization outcome in hospital *i* in county *c* in year *t*. We specifically look at outcomes such as hospital admissions, emergency room visits; inpatient days and outpatient visits; share of Medicaid and Medicare inpatient days, all of which are in logged terms. *D*_*c,t,j*_ is a vector of county-level temperature variables of interest, denoting the ten number of days in county *c* in year *t* where the daily average temperature fell in the jth of the ten temperature bins. Following Deschenes and Greenstone (2011), we create 10 temperature bins, separated by 10°F from below 10°F (−12.2°C) to above 90°F (32.2°C) and measure the number of days in a county-year with temperatures falling in each bin or prespecified degree ranges. The temperature bins of interest are the three lowest bins (below 30°F) and the two highest bins (above 80°F) as shown in Figure 2. We define hot and cold days as the following: We consider a day to be cold when the daily mean temperature falls below 30°F (−1.11°C), and a day to be hot when the mean temperature is above 80°F (26.7°C) in a given year. The omitted reference group is the sixth bin (50–60°F or 10–15.6°C). Θ is a vector of parameters of interest. For instance, the parameter for *D*_*c,t,*9_ captures how much the dependent variable changes per 10 additional days of hot days where the daily mean temperature fell in the ninth bin (*j* = 9) between 80 and 90°F (or 26.7–32.2*°*C range), relative to the impact of moderate days in 50–60°F range.

Instead of regressing health-related outcomes on mean temperature, we use ten temperature bin variables as the key independent variables. This nonparametric approach allows us to exploit the annual variation in the number of extreme hot and cold days within a county and capture the potential non-linear relationship between temperature and dependent variables without requiring strong assumptions about the functional form [17]. Earlier literature suggested threshold effect of heat or U shaped relationship between temperature and health outcomes [4, 12, 35, 38, 39].

Variable *M*_*s,t*_ is an indicator for whether state *s* where a county *c* is located, expanded their State Medicaid program under the Affordable Care Act in year *t*. All models include a vector of one-year lagged county-level characteristics that vary across time, *X*_*c,t−*1_, which include annual total precipitation, percent share of aged, black, Hispanic population, urban indicator, log of total employment and log of private-sector wage *per capita*. We also include hospital-fixed effects and year-fixed effects. Standard errors are clustered by county since average temperature varies at the county level.

To examine the impact of extreme temperature on publichealth insurance program transfers, we use a county-by-year panel and a lagged dependent variable model which is specified as Equation 2:

$$Y_{c,t}={\Theta D}_{c,t,j}+{\Phi D}_{c,t-1,j}+{\beta Y}_{c,t-1}+{\alpha M}_{s,t}+{\Pi X}_{c,t-1}+\delta c+\lambda t+\upsilon_{c,t}$$ (2)

Where *Y*_*c,t*_ measures the average level of Medicaid or Medicare benefit transfers (in log of *per capita* dollars) for county *c* in year *t*. We also include the county-fixed effects to control for all time-invariant cross-county heterogeneity and year-fixed effects to absorb annual shocks that commonly affect all counties each year. We also include *Y*_*c,t−*1_ on the righthand side of the equation to account for autocorrelation in fiscal outcomes, potential lagged effects of weather as well as unobserved time-varying confounders [35]. We also check for potential lagged effects of extreme temperature on Medical transfers, given that there may be a time lag between the day of health service and when payments from the government to vendors or intermediaries are made. In an alternative specification, we account for a potential one-year lag by including a vector of temperature variables from the previous year as regressors. We do not anticipate lagged effects when using annual data to analyze the hospital utilization effects. Previous epidemiology studies using daily or weekly data and found that delayed harvesting effects typically fade within 15–20 days [38, 40–44]. Since we use annual data, we expect health utilization behaviors to show in the contemporaneous year.

## Results

### Hospital Utilization Results

First, we find that extreme heat increases overall hospital utilization. Our estimates in Table 3 show that hot days with a daily average temperature between 80 and 90°F increase hospital admissions, inpatient days, and ER visits. Our estimates suggest that ten additional days of extreme heat, with a daily average temperature above 90°F are associated with a 1.56 percent increase in hospital admissions and 1.32 percent in inpatient days. These translate into approximately 36.5 additional admissions at hospitals (at the mean value of 2,321) and 164 more inpatient days (at the mean 16,155). Heat also increases inpatient care utilization at hospitals among patients covered by Medicaid and Medicare, as shown in the last two columns in Table 3. Ten additional days of extreme heat above 90°F increase inpatient days by 4.39 percent (91.48 at the mean 2,038) among Medicaid patients and 3.43 percent (226.91 at the mean 6,502) among Medicare patients. Ten additional hot days between 80 and 90°F are associated with a 1.69 percent (110.83 at the mean) increase in inpatient care utilization among Medicare patients.

**TABLE 3:** Impact of average daily temperature on hospital utilization (United States. 2019).

Daily temperature	Total				Medicaid	Medicare
	Admissions Inpatient days Outpatient visits ER visits				Inpatient days	Inpatient days
	(1)	(2)	(3)	(4)	(5)	(6)
Below 10°F	0.0030	0.0063	−0.0142	−0.0110**	0.0056	0.0111
	(0.004)	(0.005)	(0.011)	(0.005)	(0.013)	(0.010)
10°F–20°F	0.0158***	0.0132***	−0.0111	−0.0024	0.0295**	0.0310***
	(0.005)	(0.005)	(0.012)	(0.004)	(0.014)	(0.012)
20°F–30°F	0.0032	0.0032	−0.0057	0.0053	−0.0094	0.0092
	(0.003)	(0.003)	(0.010)	(0.004)	(0.010)	(0.008)
80°F–90°F	0.0109***	0.0101***	−0.0133	0.0068**	0.0120	0.0169***
	(0.002)	(0.003)	(0.008)	(0.003)	(0.009)	(0.006)
Above 90°F	0.0156**	0.0127*	0.0073	0.0185	0.0439**	0.0343***
	(0.007)	(0.007)	(0.022)	(0.013)	(0.020)	(0.012)
Medicaid Expansion	−0.0046	−0.0264**	−0.0326*	0.0163	0.1087***	−0.0224
	(0.010)	(0.011)	(0.017)	(0.012)	(0.029)	(0.020)
Mean Dependent variable	7.75	9.69	10.27	9.52	7.62	8.78
Observations	49,552	49,552	49,552	49,552	49,552	49,552
R-squared	0.974	0.965	0.905	0.959	0.907	0.928

Note: All models include county covariates, hospital fixed effects, year fixed effects, and county covariates. ER visits refer to emergency room visit. All hospital outcomes are in logged values. Starred entries indicate significance levels at 0.1*, 0.05**, and 0.01***.

We find that cold temperature leads to higher hospital admissions and inpatient care utilization. Ten more cold days (10–20°F) increases hospital admissions and inpatient days by 1.58 percent (36.97 at the mean) and 1.32 percent (214.66 at the mean), respectively. Notably, cold temperature has a particularly strong and positive impact on inpatient care utilization among Medicaid and Medicare patients. The latter finding is also consistent with the earlier empirical literature showing higher mortality rates among the elderly population (*≥*65Y) in the same temperature bin [4].

On the other hand, estimates in Panel A column (4) shows that extremely cold temperature below 10°F significantly reduces ER visits. Heat and cold seem to have opposite effects on ER visits. Our findings of decline in ER visits due to cold weather is similar to Davis et al. (2020), which report an immediate reduction in risks of emergency department visits at temperatures below 10°F in Virginia, explained by people being less willing to seek medical care or less likely to be outdoors.

### Medicaid and Medicare Transfer Results

Next, we examine the temperature effect on Medicaid and Medicare benefit transfers and report our estimates in Table 4. We find consistent evidence of heat increasing Medicaid transfer payments. The magnitude is largest above 90°F, where ten more days of extreme heat increases annual Medicaid transfer payments by 0.85 percent (equivalent to $11.78 *per capita*). Meanwhile, estimates in Table 4 column (1) suggest that Medicaid payments increase with mildly cold (20–30°F) days but decrease with more extremely cold days. We find that ten additional days of mild cold weather increase annual Medicaid transfers by 0.51 percent ($7.06 *per capita*).

**TABLE 4:** The impact of average daily temperature on Medicaid and Medicare transfer (United States. 2019).

Daily temperature	Baseline		With lagged temperature	
	Medicaid (1)	Medicare (2)	Medicaid (3)	Medicare (4)
Below 10°F	−0.0018*	0.0022***	−0.0019*	0.0024***
	(0.001)	(0.001)	(0.001)	(0.001)
10°F–20°F	−0.0002	−0.0005	0.0001	0.0001
	(0.001)	(0.001)	(0.001)	(0.001)
20°F–30°F	0.0051***	0.0001	0.0051***	0.0004
	(0.001)	(0.001)	(0.001)	(0.001)
80°F–90°F	0.0011*	−0.0003	0.0014**	0.0001
	(0.001)	(0.000)	(0.001)	(0.001)
Above 90°F	0.0085***	−0.0015	0.0089***	−0.0014
	(0.001)	(0.001)	(0.001)	(0.001)
Medicaid Expansion	0.0878***	0.0118***	0.0873***	0.0102***
	(0.003)	(0.001)	(0.003)	(0.001)
Mean Dependent Variable	7.23	7.58	7.23	7.58
Observations	57,799	57,809	57,678	57,688
R-squared	0.975	0.975	0.975	0.975

Note: All models include 1 year lagged dependent variable, county covariates, county fixed effects, year fixed effects and State Medicaid expansion indicator. Estimates in columns (1) and (2) are from baseline models only include contemporaneous temperature variables, while the results in columns (3) and (4) are estimated from Equation 2 which controls for one-year lagged temperature variables. Starred entries indicate significance levels at 0.1*, 0.05**, and 0.01***.

Temperature affects Medicare benefit transfer differently from how it impacts Medicaid transfer. Column (2) in Table 4 shows that ten additional days with *<*10°F increase Medicare transfer by 0.22 percent ($4.30 *per capita*). However, heat surprisingly does not seem to affect Medicare benefit transfer payments. Estimates in columns (3) and (4) report estimates from Equation 2 that include one-year lagged temperature variables on the right-hand side of the model. The contemporaneous temperature effect estimates do not change when accounting for potential lagged effects on medical benefit transfer. The coefficient estimates for the previous year’s temperature variables are all insignificant and not reported. Overall, only extreme cold (*<*10°F) seems to incur additional fiscal costs on Medicare while Medicaid costs increase due to mild cold and heat. Across all columns, we show that Medicaid expansion is positively associated with Medicaid and Medicare benefit transfers.

### Heterogeneity Analysis

In Table 5 Panel A, we report our heterogeneity tests of how Medicaid and Medicare benefit transfer varies by the average generosity of State Medicaid, which is assessed based on the information on each state’s average income eligibility thresholds (measured as the percentage of the federal poverty line) for children and parents during the sample period. The high generosity indicator equals one for a state with an average income eligibility threshold above the national median.

**TABLE 5:** The impact of average daily temperature on public medical benefit transfer: Heterogeneity by state Medicaid generosity (United States. 2019).

Panel A. Medicaid and Medicare benefit transfer		
Daily temperature	Medicaid transfer (1)	Medicare transfer (2)
Below 10°F × Low	0.0007	−0.0017
	(0.001)	(0.001)
10°F–20°F × Low	−0.0063***	0.0016
	(0.002)	(0.001)
20°F–30°F × Low	0.0004	−0.0000
	(0.001)	(0.001)
80°F–90°F × Low	−0.0028***	−0.0003
	(0.001)	(0.001)
Above 90°F × Low	0.0058***	−0.0015
	(0.001)	(0.001)
Below 10°F × High	−0.0018	0.0072***
	(0.002)	(0.001)
10°F–20°F × High	0.0146***	−0.0044***
	(0.002)	(0.001)
20°F–30°F × High	0.0121***	0.0001
	(0.002)	(0.001)
80°F–90°F × High	0.0122***	−0.0004
	(0.001)	(0.001)
Above 90°F × High	0.0177**	−0.0052
	(0.008)	(0.007)
Medicaid Expansion	0.0865*** (0.003)	0.0109*** (0.001)
Observations	57,799	57,809
R-squared	0.976	0.975

Panel B. Temperature effect on Medicare enrollment and dually eligible individuals for Medicare and Medicaid: Heterogeneity by state Medicaid generosity			
Daily temperature	Medicare enrollment	Dually eligible beneficiaries	
	Total (1)	All dual (2)	Full medicaid (3)
Below 10°F × Low	0.0008	−0.0120**	−0.0142***
	(0.002)	(0.005)	(0.005)
10°F–20°F × Low	−0.0018	−0.0018	0.0013
	(0.002)	(0.005)	(0.005)
20°F–30°F × Low	−0.0062***	−0.0040	−0.0082**
	(0.001)	(0.003)	(0.004)
80°F–90°F × Low	−0.0060***	−0.0115***	−0.0109***
	(0.001)	(0.002)	(0.002)
Above 90°F × Low	−0.0024	−0.0286***	−0.0392***
	(0.003)	(0.004)	(0.005)
Below 10°F × High	−0.0092*** (0.003)	0.0056*** (0.002)	0.0040* (0.002)
10°F–20°F × High	0.0019	−0.0014	−0.0075***
	(0.003)	(0.003)	(0.003)
20°F–30°F × High	−0.0049***	−0.0095***	−0.0116***
	(0.002)	(0.002)	(0.002)
80°F–90°F × High	−0.0002	0.0047**	0.0032
	(0.002)	(0.002)	(0.002)
Above 90°F × High	−0.0112***	0.0091*	0.0285***
	(0.003)	(0.005)	(0.007)
Medicaid Expansion	−0.0145** (0.006)	0.0679*** (0.007)	0.0960*** (0.007)
Mean Dep. Ver	8.10	6.74	4.39
Observations	39,777	39,777	39,777
R-squared	0.987	0.989	0.988

Note: All models include 1 year lagged dependent variable, county covariates, county fixed effects, year fixed effects and State Medicaid expansion indicator. Estimates in columns (1) and (2) are from baseline models only include contemporaneous temperature variables, while the results in columns (3) and (4) are estimated from Equation (2) which controls for one-year lagged temperature variables. High indicator is a dummy variable equal to one if a county is in a state with Medicaid income eligibility threshold for children and parents that is higher than the national median. The key variables of interest are the interaction terms between high (or low) dummy indicator and each temperature bin variable. Starred entries indicate significance levels at 0.1*, 0.05**, and 0.01***.

Note: All estimates are from county-annual level estimation and dependent variables are logged values of beneficiary count. High indicator is a dummy variable equal to one if a county is in a state with Medicaid income eligibility threshold for children and parents that is higher than the national median. All models include one-year lagged dependent variable, lagged county covariates, county fixed effects, year fixed effects. Starred entries indicate significance levels at 10 percent*, 5 percent**, and 1 percent***.

Estimates in column (1) indicate that extreme heat (>90°F) causes a larger increase in Medicaid benefit transfers in counties with more generous State Medicaid programs, which is consistent with our expectation Ten more hot days (80–90°F) increases Medicaid transfer by 1.2 percent in counties with more generous State Medicaid but decreases transfer by 0.28 percent in counties with less generous Medicaid. This may suggest that extreme temperatures may discourage healthcare utilization in areas with less generous State Medicaid. We also find a similar impact of cold temperatures (10–20°F) having the opposite impact on Medicaid transfer, depending on the generosity of State Medicaid. These findings coupled with a significant positive correlation (0.084, p-value of 0.047) between personal income *per capita* and State Medicaid eligibility threshold, suggest that individuals in poor areas are not receiving as much support from public health insurance programs as those living in more affluent areas. This implies a possible gap between the need for assistance and the actual provision of government welfare support.

The results in Column 2 in Table 5 Panel A show that extreme cold below 10°F also increases Medicare benefit transfer in states with more generous Medicaid, most likely explained by low-income elderly individuals who are dually enrolled in both Medicaid and Medicare. Table 5 Panel B shows whether the effects of temperature on Medicare enrollment and low-income Medicare beneficiaries are different between counties with different State Medicaid generosity. The first column shows extreme temperatures reduce total Medicare enrollment. Results in the last two columns present a positive correlation between dually eligible beneficiaries and extreme temperatures (*<*10°F and *>*80°F) among counties with more generous Medicaid, whereas participation in Medicaid among Medicare beneficiaries declines with extreme temperatures in states with less generous Medicaid. These findings imply that the generosity of the Medicaid program can have important spillover effects on Medicare costs as well.

## Discussion

In this study, we examine whether and how extreme temperature affects hospital utilization and public health insurance program spending in the United States. We focus on how hot and cold days impact healthcare utilization among low-income and elderly populations covered by Medicaid and Medicare, as this group is also particularly vulnerable to extreme weather. We find that hot and cold days increase admissions and inpatient care service utilization at hospitals, and these effects are stronger in inpatient care utilization among both Medicaid and Medicare patients. Medicaid and Medicare inpatient days increase with more hot days, while we find weaker evidence of cold weather impacting Medicaid patients’ hospital utilization. These results are consistent with findings from Illinois [45] and Virginia [14] in the United States as well as the United Kingdom [8]. Part of these findings correspond to changes in Medicaid and Medicare benefit transfers associated with extreme temperatures. We find that both mild cold (20–30°F or −6.7 to −1.11°C) and heat (*>*80°F or 26.7°C) significantly raise Medicaid transfer payment, while only extreme cold (*<*10°F or −12.2°C) increases Medicare transfer. Meanwhile, we observe a considerable heterogeneity in temperature’s effect on both medical transfers, based on median income, the share of the elderly population, and State Medicaid generosity.

The results of our paper entail important policy implications. First, this study suggests that the additional costs of extreme temperature-associated healthcare utilization may be considerable, particularly among the vulnerable population. Our results in the Supplementary Appendix show stronger positive impact of extreme temperatures on hospital utilization and medical transfers in counties with a higher share of lower-income and elderly populations. Given that Medicaid covers over 40% of all births in the U.S. and 55% of Medicaid benefit payments finance health services among the elderly and disabled adults, extreme temperatures can considerably raise Medicaid costs. In countries where the government plays both the roles of financer and insurer, these fiscal costs may vary depending on systematic features of the public health insurance system such as the eligibility criteria, types of services covered and the level of benefits.

On the other hand, it is also important to understand the fiscal implications of more generous public health policy. We find that Medicaid expansion overall has a significantly strong and positive relation with Medicaid and Medicare benefit transfers. We also find larger increases in Medicaid transfers associated with mild cold and heat among areas with more generous eligibility criteria for Medicaid. The additional fiscal costs of extreme temperature could be even higher than our estimates in countries with universal public health insurance such as Canada or South Korea, or other countries with more comprehensive public health systems where governments highly subsidize healthcare costs.

Third, our findings resonate with prior research from Mexico which shows that the national expansion of universal healthcare policy prevented cold-induced deaths [38]. This suggests that public health insurance can play a crucial role in mitigating the adverse impact of extreme weather events. Future research will be needed to advance our understanding about the roles of different public health insurance systems in coping with climate change related extreme weather events.

At the same time, it is also crucial to understand the fiscal implications of expanding public health insurance coverage. We note that the additional fiscal costs of extreme temperature could be even higher than our estimates in countries with universal public health insurance such as Canada or South Korea, or other countries with more comprehensive public health systems where governments highly subsidize healthcare costs. Future research will be needed to advance our understanding about the roles of different public health insurance systems in coping with climate change related extreme weather events.

Lastly, we have to acknowledge the limitations of this study. First of all, we cannot observe detailed service utilization other than inpatient hospital care among Medicaid patients to assess whether there were changes in primary care utilization and the nature of ER visits. Similarly, we do not observe the public health insurance transfers data at the individual level which limits our ability to discuss the equity implications. One study from the United States for instance, suggests that the mean costs of additional hospitalization due to heat is higher among racial/ethnic minorities than whites [46]. Another limitation of this study is that we cannot provide explicit policy implications about how public health insurance programs may alleviate the adverse impact of extreme weather. Our findings suggest that if more people use health services due to extreme temperatures, expanding public health insurance coverage could encourage vulnerable populations to utilize necessary services by lowering their out-of-pocket payments. These can be subjects for future research.
