# Supplementary figures and images for: Extreme Temperatures, Hospital Utilization and Public Health Insurance Spending
Source: Int J Public Health. 2025 Feb 12;70:1607160. doi: 10.3389/ijph.2025.1607160 (PMC11859587; doi:10.3389/ijph.2025.1607160)

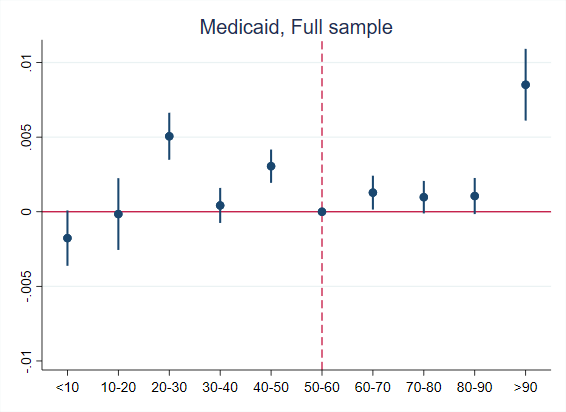

Supplement: Supplementary file 1 [file Image5.PNG]

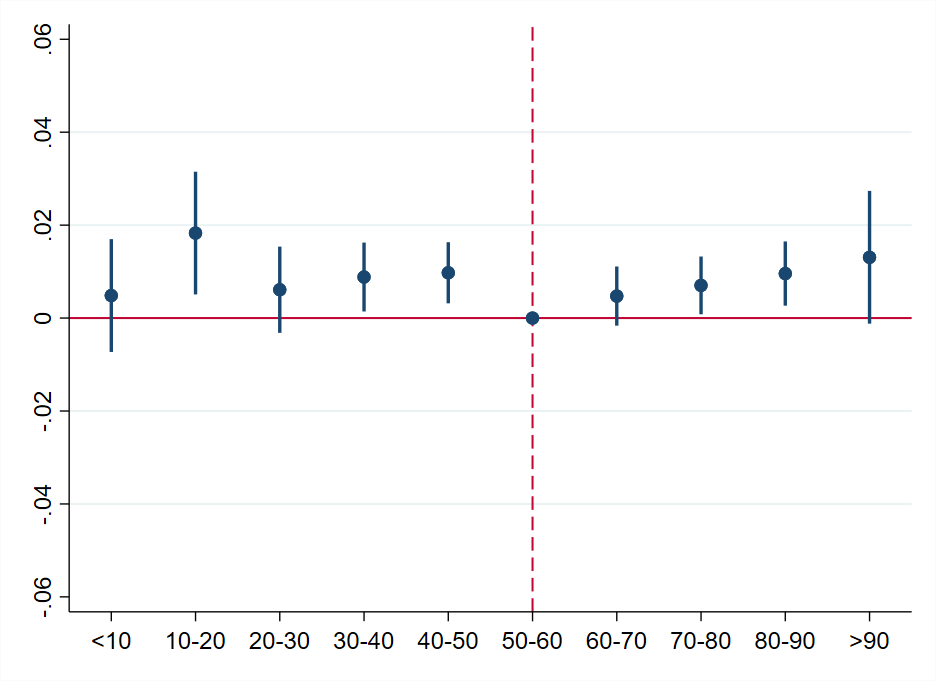

Supplement: Supplementary file 2 [file Image4.PNG]

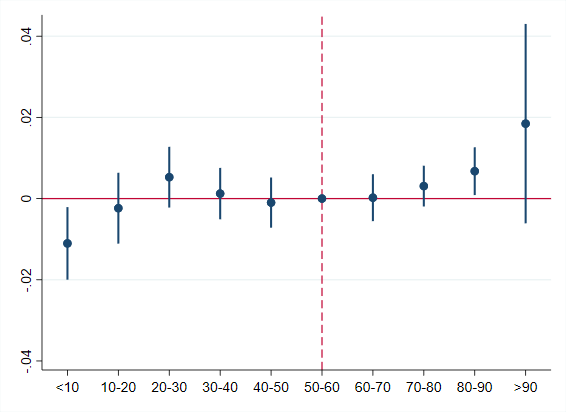

Supplement: Supplementary file 4 [file Image2.PNG]

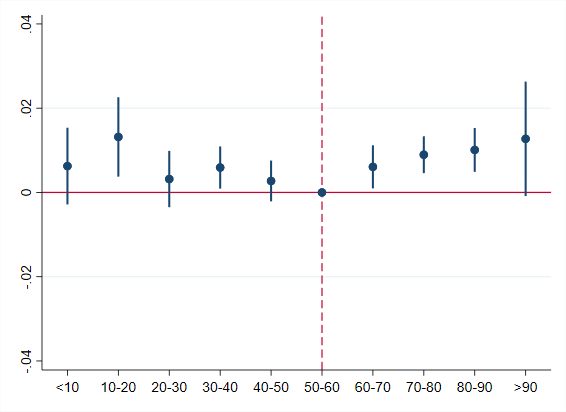

Supplement: Supplementary file 5 [file Image1.PNG]

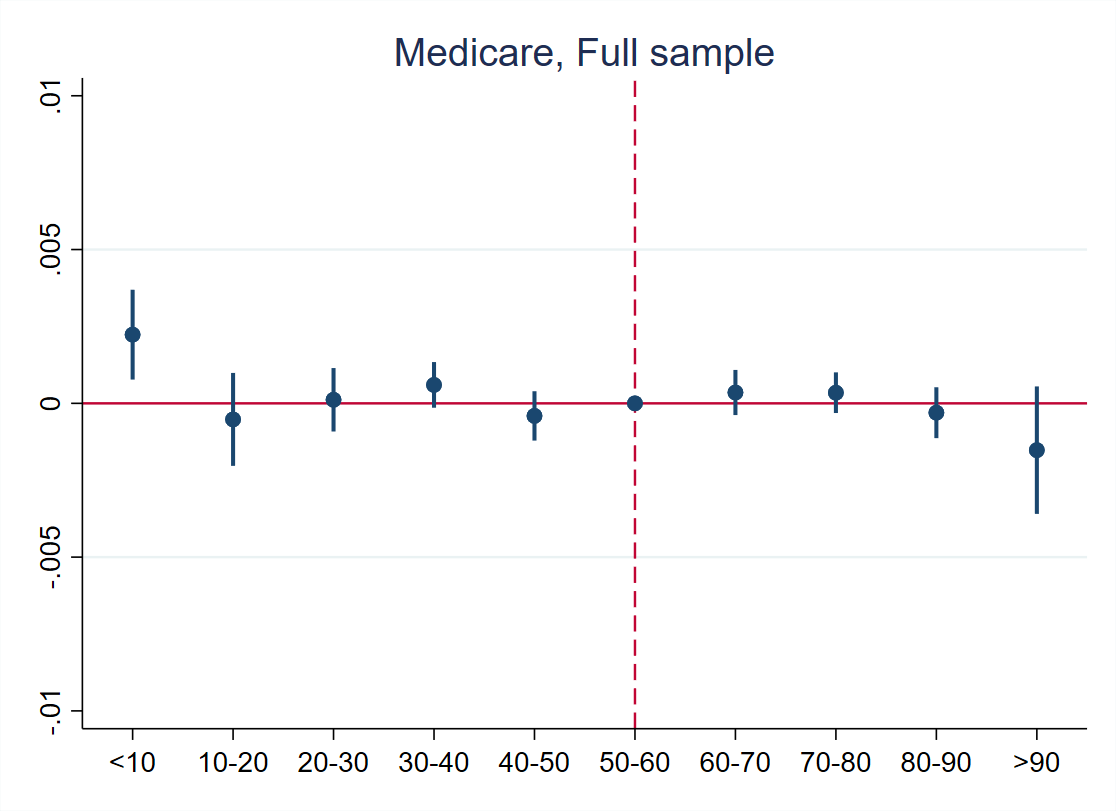

Supplement: Supplementary file 6 [file Image6.PNG]

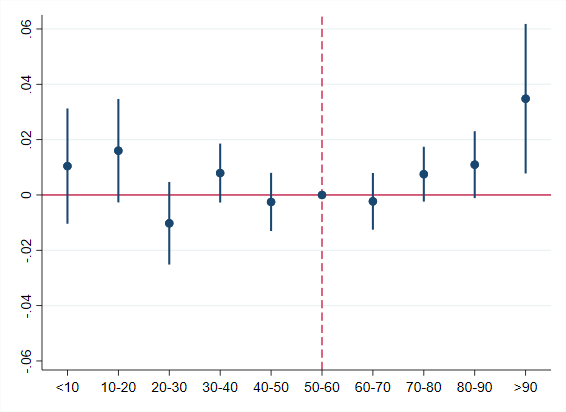

Supplement: Supplementary file 7 [file Image3.PNG]
