# Supplementary material for: Extreme Temperatures, Hospital Utilization and Public Health Insurance Spending
Source: Int J Public Health. 2025 Feb 12;70:1607160. doi: 10.3389/ijph.2025.1607160 (PMC11859587; doi:10.3389/ijph.2025.1607160)
Supplement: Supplementary file 3 [file DataSheet1.pdf]

Appendix

As an extension, we examine the heterogeneity of the temperature impacts on hospital utilization and public medical transfer, based on the county income levels and the share of the elderly population. Specifically, we divide our estimation sample into two groups based on whether the county-level personal income and the percentage of the senior population were above or below the national median, in baseline year 2000. Then, we interact the temperature variables with both "High" and "Low" indicators to directly compare the temperature impacts in the two groups simultaneously. The idea is to test whether extreme weather more significantly increases the utilization of hospitals that serve more socially vulnerable populations. Our heterogeneity results also suggest how vulnerable populations may be more susceptible to extreme heat. Results in Appendix Table A1 columns (1) and (3) shows that counties with lower income experience significant increases in admissions and inpatient care at hospitals when there are more hot days above 90°F. We find a similar pattern in counties with a higher share of the elderly population from columns (2), (4) and (6): Extreme heat above 90°F is positively correlated with hospital admissions, inpatient days, and ER visits in counties with more elderly.

We also find a similar pattern for inpatient care utilization among Medicaid and Medicare patients. Estimates in column (8) show that extreme heat increases Medicaid inpatient days in counties with more elderly. Results in column (9) report consistent evidence of a significant increase in Medicare inpatient days in response to cold and extreme heat in lower-income counties. Column (10) presents a noticeable positive association between Medicare inpatient days and temperature extremes in counties with more elderly. Our findings are consistent with previous studies that demonstrated a heat-related increase in hospitalization among people at or over the age of 65 (Anderson et al. 2013; Gronlund et al. 2016).

Results in Table A1 column (6) suggest that the drop in ER visits may be mostly driven

by areas with fewer elderly. We also do not observe a significant change in ER visits among lower-income counties associated with cold temperatures, while ER visits increase among relatively more affluent counties when there are more cold days with 20 to 30°F. This is particularly concerning if low-income individuals consider emergency rooms as an entry point into the healthcare system. Cheung et al. (2012), for instance, illustrate how low-income Medicaid beneficiaries in general have higher utilization of ER services (39.6 v.s. 17.7 percent) and more barriers to primary care than individuals covered by private health insurance. If cold weather limits either access to or willingness to seek necessary emergency care among poor individuals, delays in medical care can exacerbate severe health conditions that eventually require hospitalization. If there are pre-existing inequities in access to healthcare service across different income groups the relationship between extreme temperature and outcomes (public health insurance cost and health utilization) may be different between relatively more affluent and poor counties. We test for this by comparing counties that have county-area-personal income that is higher than the national median and those that have lower income. Indeed, we find significantly higher hospital admissions and inpatient service utilization among lower-income counties when there are more cold days of temperatures below 30 ◦F. The insignificant changes in Medicaid transfer and ER visits among lower-income counties raise an equity concern about whether extreme temperature disrupts access to healthcare. This is particularly puzzling given that several studies report high infant and elderly mortality rates during hot days (Braga et al. 2001; Deschenes and Greenstone 2011). Altogether our findings beg the question of whether extreme weather further limits access to health care in lower-income areas which may disproportionately affect the health of disadvantaged individuals. The estimates in Appendix Table A2 suggest that the extreme cold-induced increase in Medicare transfer is mostly driven by counties with higher income and fewer elderly (or a relatively lower share of vulnerable populations based on income and age). On the other hand, heat reduces Medicare transfer in counties with a higher share of the elderly population. This may at first seem puzzling given our

findings from Table 3 column (6) where we find heat positively associated with inpatient care utilization among Medicare patients. However, Appendix Table A1 shows that Medicare inpatient care costs decrease with more hot days (80-90°F), which may be due to changes in the types of treatments or other supply-side factors. Another explanation for the reduction in Medicare transfer is the potential decline in outpatient service utilization. We find that hot days with daily temperatures at 80-90○F reduce outpatient hospital visits in counties with more elderly people. Although we do not have data on outpatient service utilization among Medicare patients, the majority of Medicare benefit spending goes to Part B service (which includes outpatient services, physician-administered drugs, or primary care visits). Medicare Part B spending accounted for 48 percent of total Medicare benefit expenditure in 2021, according to the Centers for Medicare & Medicaid Services. Alternatively, a higher risk of mortality among the elderly population may also contribute to the reduction in outpatient services among Medicare beneficiaries.

Columns 3 and 4 in Table A2 present the heterogeneity effect estimates on Medicare benefit transfer. The estimates suggest that the extreme cold-induced increase in Medicare transfer is mostly driven by counties with higher income and fewer elderly (or a relatively lower share of vulnerable populations based on income and age). On the other hand, heat reduces Medicare transfer in counties with a higher share of the elderly population. This may at first seem puzzling given our findings from Table 2 column (6) where we find heat positively associated with inpatient care utilization among Medicare patients. However, we find that Medicare inpatient care costs decrease with more hot days (80-90°F), which may be due to changes in the types of treatments or other supply-side factors. Another explanation for the reduction in Medicare transfer is the potential decline in outpatient service utilization. We find that hot days with daily temperatures at 80-90°F reduce outpatient hospital visits in counties with more elderly people. Although we do not have data on outpatient service utilization among Medicare patients, the majority of Medicare benefit spending goes to

Part B service (which includes outpatient services, physician-administered drugs, or primary care visits). Medicare Part B spending accounted for 48 percent of total Medicare benefit expenditure in 2021, according to the Centers for Medicare & Medicaid Services. Alternatively, a higher risk of mortality among the elderly population may also contribute to the reduction in outpatient services among Medicare beneficiaries. While we cannot infer how the elderly may respond to extreme weather relative to younger people, the reduction in outpatient services among Medicare beneficiaries could potentially be related to a higher likelihood of staying at home. Overall, our findings provide distributional implications regarding access to care among vulnerable populations as well as what roles the existing public health insurance programs play in extreme weather conditions.

Appendix Table A1. Heterogeneity effect estimates: Hospital utilization

	All patients						Medicaid and Medicare patients			
	Admissions		Inpatient days		ER visits		Medicaid Inpatient days		Medicare Inpatient days	
Daily temperature	Income (1)	Elderly (2)	Income (3)	Elderly (4)	Income (5)	Elderly (6)	Income (7)	Elderly (8)	Income (9)	Elderly (10)
Below 10°F x Low	0.0004	-0.0039	0.0011	0.0062	-0.0130	-0.0326**	-0.0020	0.0080	0.0098	0.0148
	(0.004)	(0.006)	(0.006)	(0.006)	(0.009)	(0.016)	(0.015)	(0.020)	(0.010)	(0.017)
10-20°F x Low	0.0239***	0.0102	0.0237***	0.0070	0.0017	-0.0079	0.0415**	0.0106	0.0250**	0.0016
	(0.006)	(0.008)	(0.007)	(0.008)	(0.010)	(0.018)	(0.016)	(0.020)	(0.012)	(0.017)
20-30°F x Low	0.0086**	-0.0056	0.0102**	-0.0034	0.0049	0.0008	0.0046	-0.0234*	0.0247***	-0.0023
	(0.004)	(0.004)	(0.004)	(0.004)	(0.007)	(0.013)	(0.012)	(0.013)	(0.008)	(0.011)
80-90°F x Low	0.0066***	0.0101***	0.0061*	0.0104***	0.0073	-0.0027	0.0106	0.0091	0.0107*	0.0158**
	(0.003)	(0.003)	(0.003)	(0.003)	(0.009)	(0.010)	(0.010)	(0.011)	(0.006)	(0.008)
Above 90°F x Low	0.0347***	0.0015	0.0262***	0.0019	0.0171	0.0097	0.0777***	0.0144	0.0475***	0.0179
	(0.006)	(0.006)	(0.008)	(0.007)	(0.014)	(0.017)	(0.022)	(0.020)	(0.014)	(0.013)
Below 10°F x High	0.0027	0.0067	0.0137**	0.0063	-0.0140	-0.0059	0.0208	0.0058	0.0043	0.0096
	(0.007)	(0.004)	(0.006)	(0.006)	(0.018)	(0.010)	(0.025)	(0.016)	(0.019)	(0.011)
10-20°F x High	0.0012	0.0235***	-0.0044	0.0198***	-0.0142	-0.0005	0.0022	0.0475***	0.0350*	0.0556***
	(0.006)	(0.005)	(0.005)	(0.005)	(0.021)	(0.013)	(0.021)	(0.017)	(0.020)	(0.013)
20-30°F x High	-0.0086**	0.0106***	-0.0100***	0.0082*	0.0402***	0.0321***	-0.0402***	0.0003	-0.0225*	0.0175*
	(0.004)	(0.004)	(0.004)	(0.004)	(0.015)	(0.010)	(0.015)	(0.013)	(0.013)	(0.010)
80-90°F x High	0.0088***	0.0112***	0.0094***	0.0088***	-0.0023	0.0087	0.0012	0.0100	0.0153**	0.0157**
	(0.003)	(0.003)	(0.003)	(0.003)	(0.009)	(0.008)	(0.011)	(0.009)	(0.007)	(0.006)
Above 90°F x High	0.0000	0.0459***	0.0020	0.0365***	0.0225	0.0435**	0.0170	0.1066***	0.0241*	0.0707***
	(0.007)	(0.007)	(0.007)	(0.009)	(0.020)	(0.017)	(0.023)	(0.025)	(0.015)	(0.016)
Medicaid Expansion	-0.0121	-0.0059	-0.0326***	-0.0284***	0.0182	0.0200	0.0924***	0.1041***	-0.0348*	-0.0264
	(0.009)	(0.009)	(0.010)	(0.011)	(0.020)	(0.020)	(0.029)	(0.029)	(0.020)	(0.020)
Observations	49,552	49,552	49,552	49,552	49,552	49,552	49,552	49,552	49,552	49,552
R-squared	0.974	0.974	0.965	0.965	0.955	0.955	0.907	0.907	0.929	0.929

Note: All models include hospital fixed effects, year fixed effects and county covariates. All hospital utilization variables are in logged values. ER visits stands for Emergency Room visits.Low is a dummy indicator of counties that have lower than the national median level of county median income or share of the elderly population (≥ 65). High indicator is a dummy of counties that have higher county median income or elderly population, compared to the national median. Starred entries indicate significance levels at 10 percent*, 5 percent**, and 1 percent***.

Appendix Table A2. Temperature impact on public medical benefit transfer: Heterogeneity by median income and share of senior population

Daily temperature	Medicaid transfer		Medicare transfer	
	Median income	Senior population	Median income	Senior population
	(1)	(2)	(3)	(4)
Below 10°F x Low	-0.0003	-0.0030**	0.0002	0.0053***
	(0.001)	(0.001)	(0.001)	(0.001)
10-20°F x Low	-0.0028	-0.0005	-0.0005	-0.0004
	(0.002)	(0.002)	(0.001)	(0.001)
20-30°F x Low	0.0015	0.0030***	0.0002	0.0002
	(0.001)	(0.001)	(0.001)	(0.001)
80-90°F x Low	-0.0001	-0.0009	-0.0008	0.0009*
	(0.001)	(0.001)	(0.001)	(0.000)
Above 90°F x Low	0.0074***	0.0054***	-0.0015	-0.0001
	(0.001)	(0.002)	(0.001)	(0.002)
Below 10°F x High	-0.0021*	-0.0004	0.0034***	0.0004
	(0.001)	(0.001)	(0.001)	(0.001)
10-20°F x High	0.0019	0.0001	-0.0005	-0.0010
	(0.002)	(0.002)	(0.001)	(0.001)
20-30°F x High	0.0080***	0.0066***	0.0001	-0.0001
	(0.001)	(0.001)	(0.001)	(0.001)
80-90°F x High	0.0023***	0.0033***	0.0002	-0.0018***
	(0.001)	(0.001)	(0.001)	(0.001)
Above 90°F x High	0.0107***	0.0112***	-0.0025	-0.0028**
	(0.002)	(0.002)	(0.002)	(0.001)
Medicaid Expansion	0.0881***	0.0876***	0.0107***	0.0108***
	(0.003)	(0.003)	(0.001)	(0.001)
Observations	57,799	57,799	57,809	57,809
R-squared	0.975	0.975	0.975	0.975

Note: All models include one year lagged dependent variable, county covariates, county fixed effects, year fixed effects and State Medicaid expansion indicator. The mean values of the dependent variables in Panels A and B are 7.17 and 7.58, respectively. Starred entries indicate significance levels at 10 percent*, 5 percent**, and 1 percent***.

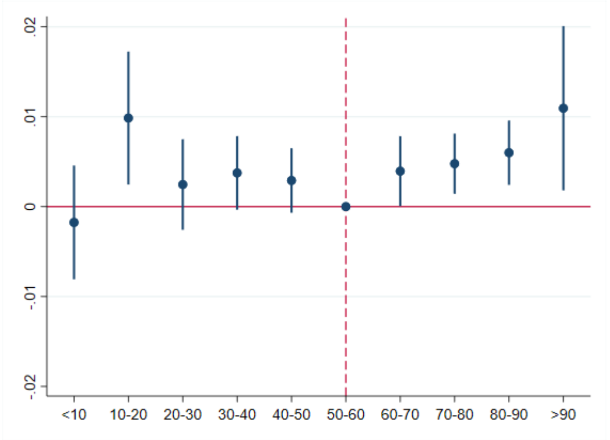

(a) Total hospital admission

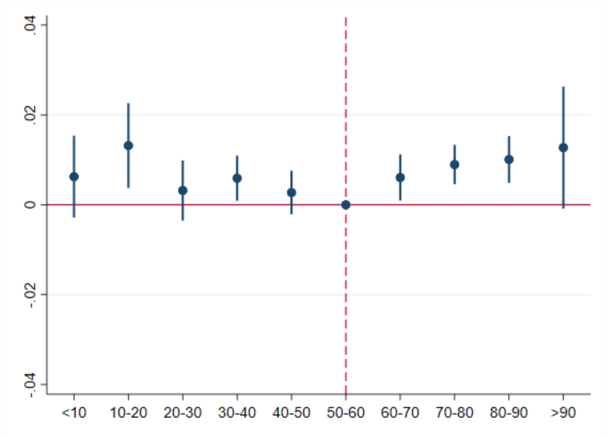

(b) Total inpatient days

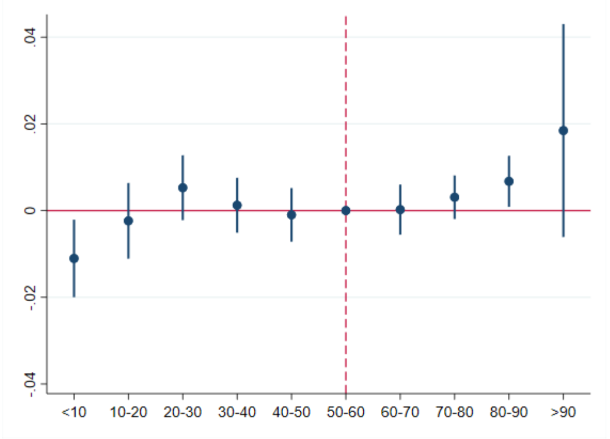

(c) Emergency Room Visits

Figure A1. Temperature effect on overall hospital utilization
Note: Each panel shows the point estimates of the impact of temperature on hospital utilization outcomes using annual hospital-level data. The horizontal axis indicates the temperature bins (in farenheit) and the vertical axis shows the size of coefficient estimates.

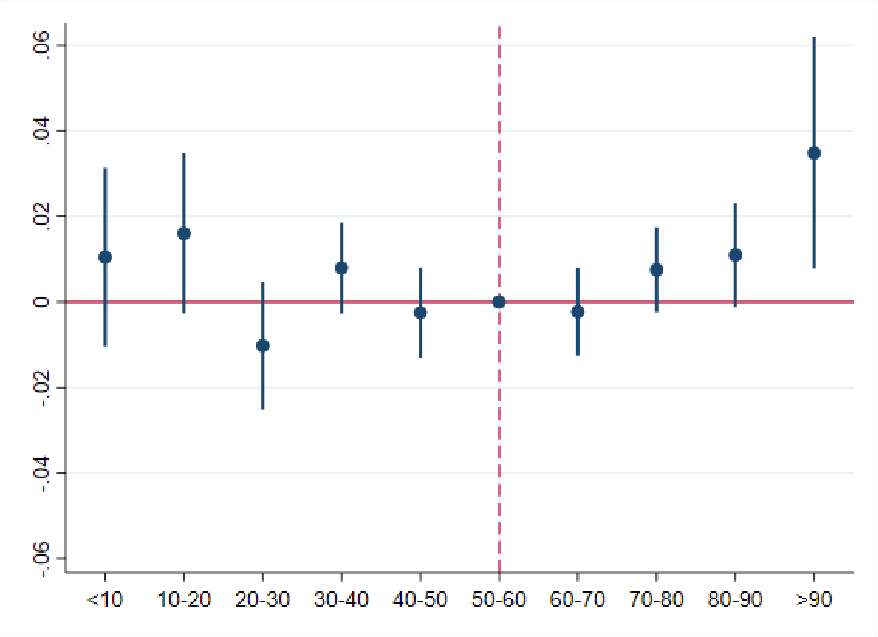

(a) Estimated effect of 10 days of average temperature on Medicaid hospital inpatient days

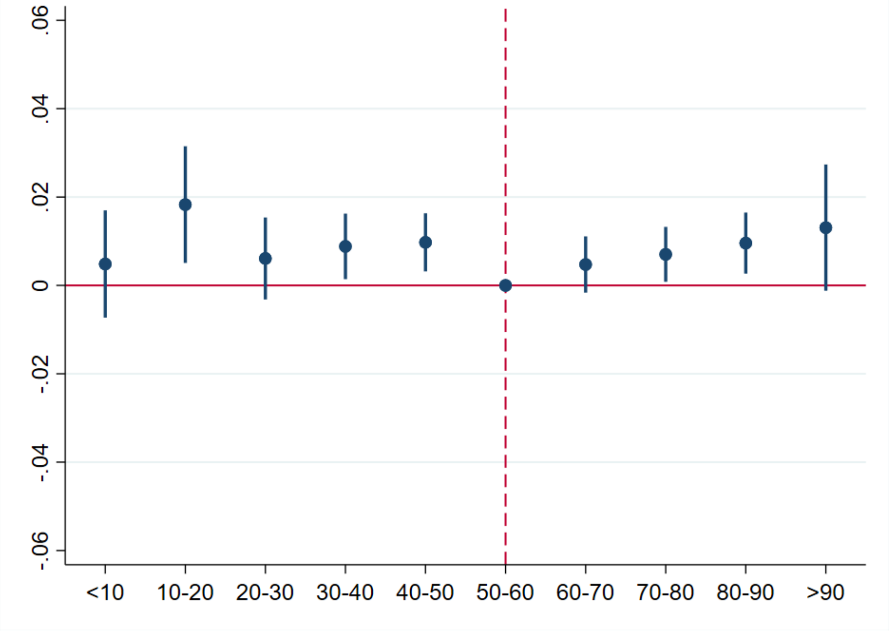

(b)Estimated effect of 10 days of average temperature on Medicare hospital inpatient days

Figure A2. Temperature effect on hospital utilization of Medicaid and Medicare patients

Note: Each panel shows the point estimates of the impact of temperature on hospital utilization outcome (log of Medicaid and Medicare inpatient days) using annual hospital-level data. The horizontal axis indicates the temperature bins (farenheit) and the vertical axis shows the size of coefficient estimates.

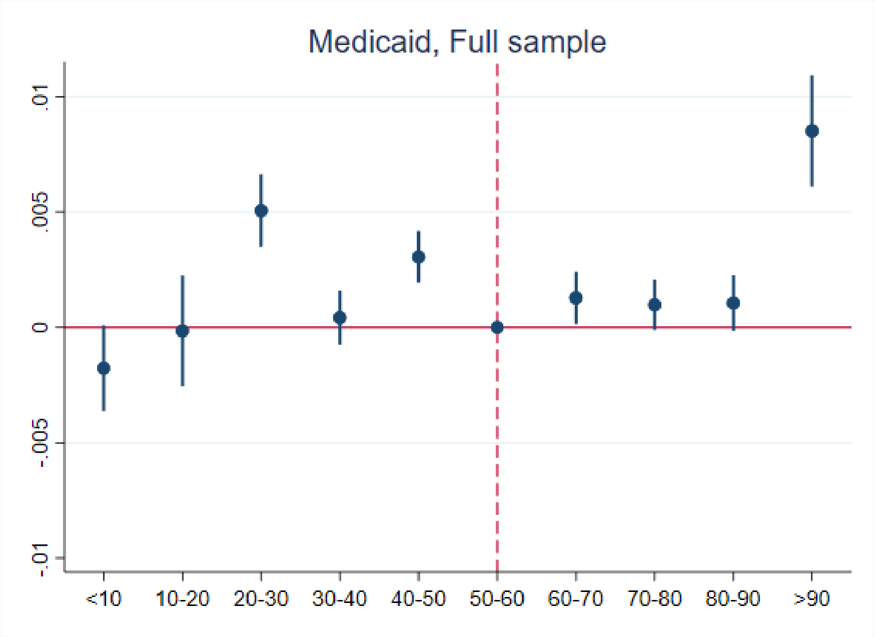

(a)

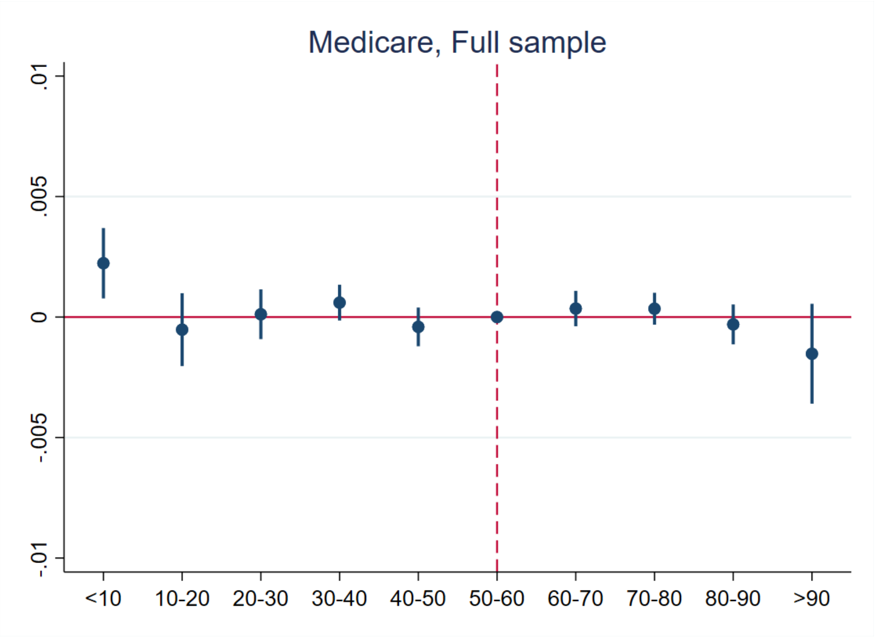

(b)

Figure A3.Temperature effect on Medicaid and Medicare transfer

Note: Each panel shows the point estimates of the impact of temperature on annual transfer payments through Medicaid and Medicare programs. The horizontal axis indicates the temperature bins (in farenheit) and the vertical axis shows the size of coefficient estimates.
